# Crocin Improved Learning and Memory Impairments in Streptozotocin-Induced Diabetic Rats

**Published:** 2013-01

**Authors:** Esmaeal Tamaddonfard, Amir Abbas Farshid, Siamak Asri-Rezaee, Shahram Javadi, Voria Khosravi, Bentolhoda Rahman, Zahra Mirfakhraee

**Affiliations:** 1 *Department of Basic Sciences, Faculty of Veterinary Medicine, Urmia University, Urmia, Iran*; 2 *Department of Pathobiology, Faculty of Veterinary Medicine, Urmia University, Urmia, Iran*; 3 *Department of Clinical Sciences, Faculty of Veterinary Medicine, Urmia University, Urmia, Iran*

**Keywords:** Crocin, Diabetic rats, Learning and memory, Oxidative stress

## Abstract

***Objective(s): ***Crocin influences many biological functions including memory and learning. The present study was aimed to investigate the effects of crocin on learning and memory impairments in streptozotocine-induced diabetic rats.

***Materials and Methods:*** Diabetes was induced by intraperitoneal (IP) injection of streptozotocin (STZ, 45 mg/kg). Transfer latency (TL) paradigm in elevated plus-maze (EPM) was used as an index of learning and memory. Plasma levels of total antioxidant capacity (TAC) and malondialdehyde (MDA), blood levels of glucose, and serum concentrations of insulin were measured. The number of hippocampal neurons was also counted.

***Results: ***STZ increased acquisition transfer latency (TL_1_) and retention transfer latency (TL_2_), and MDA, decreased transfer latency shortening (TLs) and TCA, produced hyperglycemia and hypoinsulinemia, and reduced the number of neurons in the hippocampus. Learning and memory impairments and blood TCA, MDA, glucose, and insulin changes induced by streptozotocin were improved with long-term IP injection of crocin at doses of 15 and 30 mg/kg. Crocin prevented hippocampal neurons number loss in diabetic rats.

***Conclusion: ***The results indicate that oxidative stress, hyperglycemia, hypoinsulinemia, and reduction of hippocampal neurons may be involved in learning and memory impairments in STZ-induced diabetic rats. Antioxidant, antihyperglycemic, antihypoinsulinemic, and neuroprotective activities of crocin might be involved in improving learning and memory impairments.

## Introduction

Diabetes is a metabolic disorder characterized by hyperglycemia due to defects in insulin secretion, insulin function, or both. Chronic hyperglycemia results in dysfunction and sustained injuries in various organs such as kidney, eyes, heart and, especially nervous system (1). Besides peripheral neuropathy (2), some evidences suggest that diabetes has strong effects on neuroregulatory, motor, and cognitive functions of the brain (3-5). 

The hippocampus, as a part of limbic system, is involved in modulation of stress, anxiety, epilepsy, pain, and cognition (6-10). Cognitive impairments associated with diabetes mellitus caused by inadequate insulin/insulin receptor functions in brain have been documented (11). In addition, chronic hyperglycemia has been related to the impairment of hippocampal neurogenesis in diabetic animals (12). Oxidative stress is widely accepted as playing a key mediatory role in the development and progression of diabetes and its complications such as cognitive deficits (13-14). Moreover, lower neuronal proliferation in the hippocampus has been implicated in lower cognitive performance in rodents, mainly in memory tasks (15). 

Crocin is the major yellow pigment of saffron and gardenia yellow, which are extracts of *Crocus sativus* stigmas and* Gardenia jasminoides* fruits, respectively (16-17). Recent studies have suggested anti-inflammatory, anti-edematous, antiepileptic, analgesic, nerve regeneration enhancing, microtubule polymerization, and antioxidant properties of crocin (18-25). Additionally, recent works have shown that crocin improves chronic cerebral hypoperfusion- and chronic stress-induced learning and memory impairments through its antioxidant effect (10, 26). 

In the present study, we investigated the effects of crocin on learning and memory impairments, plasma TAC and MDA levels, blood glucose, serum insulin, and hippocampal neurons number in streptozotocin-induced diabetic rats. We used transfer latency (TL) paradigm in elevated plus maze (EPM) because it has been proposed that the TL may be useful for calculation of learning and memory functions in mice and rats (27-30). 

## Materials and Methods

***Animals***

Healthy adult male Wistar rats, weighing 220–240 g were used in this study. Rats were maintained in groups of 6 per cage in a light-dark cycle (light on at 07:00 hr) at a controlled ambient temperature (22±0.5°C) with *ad libitum* food and water. Six rats were used for each experiment. All research and animal care procedures were approved by the Veterinary Ethics Committee of the Faculty of Veterinary Medicine of Urmia University and were performed in accordance with the National Institutes of Health Guide for Care and Use of Laboratory Animals.

***Chemicals***

Crocin and streptozotocin powders were purchased from Fluka (Germany) and Sigma-Aldrich (USA), respectively. All the analytical chemicals including sodium dodecyl sulphate, acetic acid, thiobarbituric acid, n-butanol, pyridine, 2,4,6-tripyridyl-S-triazine (TPTZ), and FeCl_3_. 6H_2_O were purchased from Merck Chemical Co. ().

***Treatment groups***

In this study, 48 rats were divided into eight experimental groups as follows: citrate buffer + normal saline, citrate buffer+crocin (7.5 mg/kg), citrate buffer+crocin (15 mg/kg), citrate buffer+crocin (30 mg/kg), STZ(60 mg/kg) + normal saline, STZ (60 mg/kg) + crocin (7.5 mg/kg), STZ (60 mg/kg)+crocin (15 mg/kg), and STZ (60 mg/kg)+crocin (30 mg/kg). Normal saline and crocin were IP injected daily from 4th day after IP injection of citrate buffer and streptozotocine and lasted to the end of the experiment (30 days). In the present study, the doses of crocin were designed according to previous studies in which the used doses of crocin were 50-200 mg/kg for 5 days and 15, 30, and 5-20 mg/kg for 21 days (10, 31, 32). 

***Induction of diabetes***

Diabetes mellitus was induced in overnight fasted rats by a single IP injection of 50 mg/kg of freshly prepared STZ (33). STZ was dissolved in sodium citrate buffer (0.1 M, pH 4.5). Hyperglycemia was confirmed by the elevated glucose levels in plasma, determined at 72 hr after injection of the citrate buffer and streptozotocin, using a digital glucometer (Elegans, Germany). The animals with blood glucose concentration more than 250 mg/dl were used for the study.

***Elevated plus maze test***

Acquisition and retention memory processes were assessed using the elevated plus maze (27-30). The EPM was made of wood and consisted of two closed arms (50×10×40 cm) and two open arms (50×10 cm) forming a cross, with a quadrangular center (10×10 cm). Open arms were surrounded by a short (2 cm) plexiglass edge to prevent falls. The height of the maze was 50 cm above the floor. On the 1^st^ day (day 29 after induction of diabetes), the acquisition transfer latency (TL_1_) was carried out as follows: the rats were placed individually at the end of one open arm facing away from central platform and the time took to move from the open arm to either of the enclosed arm was recorded. The TL was the time whena rat was placed on the open arm and when all its four legs cross to the enclosed arm. In this experiment, when the rat did not enter the enclosed arm for 90 sec, it was gently pushed on the back into the enclosed arm and the transfer latency was assigned 90 sec. The rat was allowed to move freely in the plus maze regardless of open and closed arms for 20 s after the measurement of transfer latency. The rat was then gently taken out of the plus maze and was returned to its home cage. Twenty-four hours later (day 30 after induction of diabetes), the retention transfer latency (TL_2_) test was performed in the same manner as in the acquisition trial. The rats were put into the open arm and the transfer latency was recorded again. If the rat did not enter the enclosed arm within 90 s on the 2^nd^ trial, the transfer latency was assigned 90 s. Usually, there is a shortening of the TL on the second day, relative to the first, the percentage of TL shortening [(TL_1_ – TL_2_) × 100 / TL_1_] was also calculated (30). The experiments were conducted between 12:00 and 17:00 hr in a semi-soundproof room under a natural illumination. The maze was cleaned after each rat. Each rat was tested only once. 

***Blood sampling***

At the end of the experiments (day 31 after induction of diabetes), fasting blood glucose levels were measured. Then, the animals were deeply anesthetized with IP injection of a mixture of ketamine (150 mg/kg) and xylazine (15 mg/kg) and blood samples were collected from the heart into heparin and non-coagulant containing tubes to obtain plasma and serum, respectively. The plasma was separated and kept at -80ºC until analysis of TAC and MDA. Blood samples of non-coagulant containing tubes were centrifuged at 3500 rpm for 10 min and separated serum samples transferred to Eppendorf tubes and stored at -80ºC until analysis. Thereafter, the rats were euthanized using intracardiac injection of 0.5 ml thiopental sodium (Biochemie GmbH, ). Necropsy of the animals was performed and the brain was removed and fixed in 10% buffer formal saline. 

***Biochemiacal assay***

Plasma TAC was determined by measuring the ability to reduce Fe^3+^ to Fe^2+^ as named ferric reducing antioxidant power (FRAP) (34). The reagent included 2,4,6-tripyridy-S-triazine (TPTZ), FeCl_3_, and acetate buffer. Twenty microliter of water-diluted plasma was added to 600 microliter of freshly prepared reagent warmed at 37°C. The complex between Fe^2+^ and TPTZ gives a blue color with absorbance at 593 nm. Plasma TAC levels were expressed as nmol/ml.

Plasma MDA levels were measured by the thiobarbitoric (TBA) acid method which was modified from Yagi method (35). Peroxidation was measured as the production of MDA, which in combination with TBA forms a pink chromogen compound whose absorbance was measured spectrophotometrically (JASCO, UV-975, ) at 532 nm. Serum MDA results were expressed as nmol/ml.

Serum insulin concentrations were detected using an ELISA test kit after the serum samples were thawed at room temperature. This assay has a sensitivity margin of 0.5 μIU/ml. Insulin ELISA kit was obtained from DRG instruments Gmbh, , Cat no. (EIA 2935). 

***Histopathology***

The brains were routinely fixed in 10% buffered formal saline and processed for paraffin embedding. Thin sections (4-5 μm) were cut using a microtome and stained with hematoxylin and eosin (H&E) and then examined using a light microscope. According to Paxinos and Watson (36), various regions of the hippocampus were marked and the number of neurons in CA1 region of the hippocampus was counted by special morphometric lens in 0.25 mm^2 ^microscopic field, from 10 different areas of the sections and the mean values were calculated. The final number of hippocampal neurons was expressed as the mean of the number counted in six animals per group. It is well known that various region of hippocampal formation have documented roles in learning and memory functions. The CA1 region of the hippocampus is the most sensitive and the first place influenced by hazardous conditions such as diabetes (37). 

***Statistical analysis***

The values are expressed as mean±SEM of six animals. Differences between groups were assessed by one-way analysis of variance (ANOVA) followed by Duncan^,^s test. Significance at *P*<0.05 has been given receptive in the figures.

## Results

 No significant differences were observed among citrate buffer+normal saline, citrate buffer+crocin (7.5 mg/kg), citrate buffer+crocin (15 mg/kg), and citrate buffer+crocin (30 mg/kg) groups on TL_1_ and TL_2_ time durations, TLs percentage, glucose, insulin, MDA and TAC levels, and number of hippocampal neurons. The related data have not been shown in the figures. 

**Figure 1:**
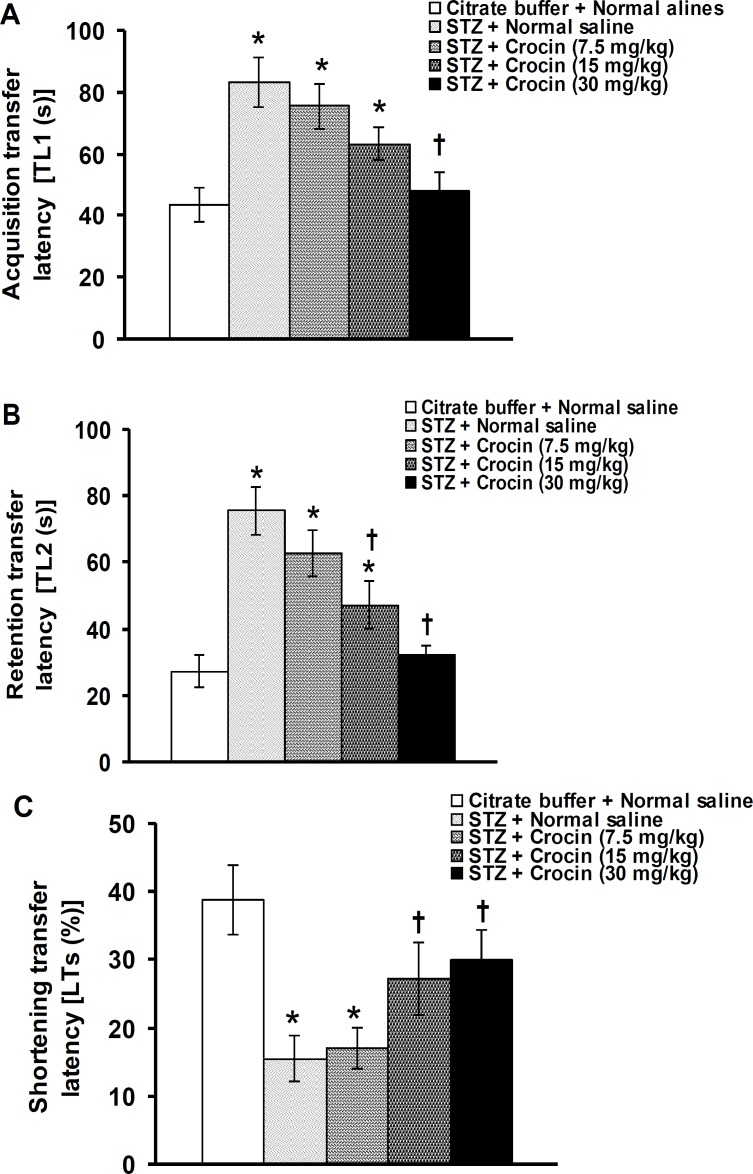
Effects of crocin on LT1 (A) and LT2 (B) time durations and LTs percentage changes induced by streptozotocin (STZ) in rats. Data are presented as mean±SEM (n= 6). *P<0.05 denotes significant difference *vs* citrate buffer + normal saline treated group. ^†^*P*<0.05 denotes significant difference *vs* STZ + normal saline treated group

Figure 1 shows the effect of crocin on TL_1_ and TL_2_ time duration and percentage of TLs changes in streptozotocin-induced diabetic rats. Streptozotocin significantly increased TL_1_ and TL_2_ time durations and decreased TLs percentage. One-way ANOVA revealed that crocin at a dose of 7.5 mg/kg produced no significant effects, whereas at doses of 15 and 30 mg/kg, crocin significantly decreased TL_1_ (F(4,25)= 6.726, *P*<0.05, Figure 1A), TL_2_ (F(4,25)= 11.334, *P*<0.05, Figure 1B) time durations and increased TLs percentage (F(4,25)= 4.787, *P*<0.05, Figure 1C) in diabetic rats. 

**Figure 2:**
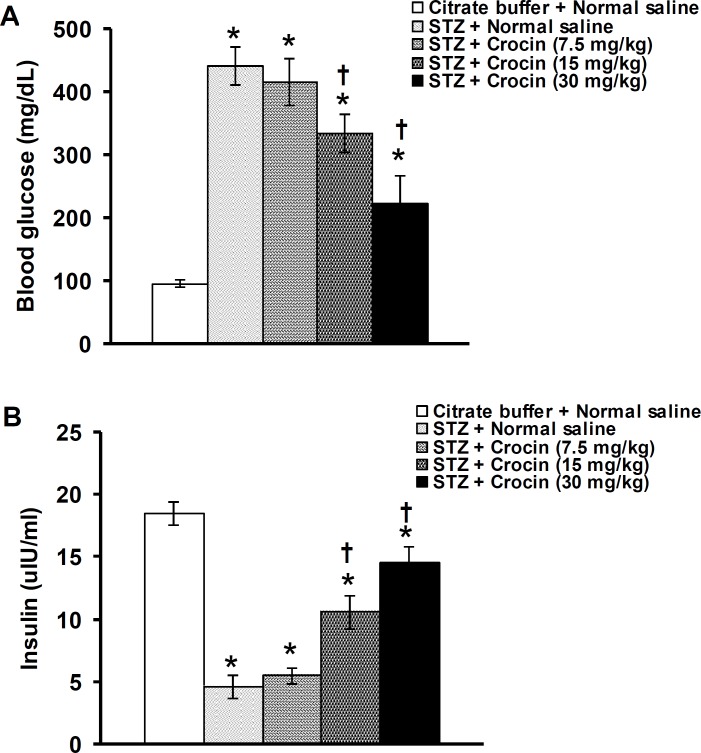
Effects of crocin on blood glucose (A) and serum insulin (B) level changes induced by streptozotocin (STZ) in rats. Data are presented as mean±SEM (n= 6). *P<0.05 denotes significant difference *vs* citrate buffer + normal saline treated group. ^†^*P*<0.05 denotes significant difference *vs* STZ + normal saline treated group

**Figure 3:**
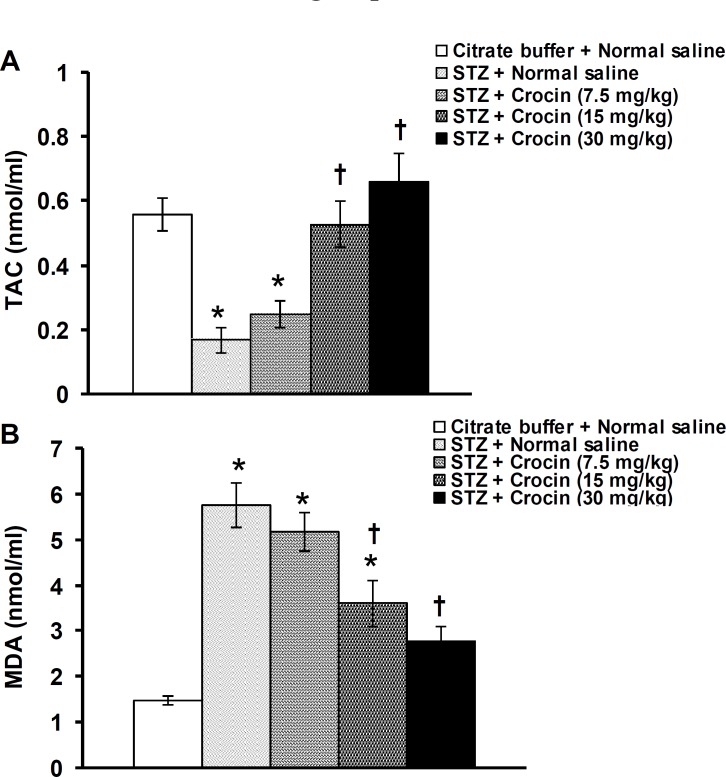
Effects of crocin on plasma TAC (A) and MDA (B) level changes induced by streptozotocin (STZ) in rats. Data are presented as mean±SEM (n= 6). *p<0.05 denotes significant difference *vs* citrate buffer+normal saline treated group. ^†^*P*<0.05 denotes significant difference *vs* STZ + normal saline treated group

**Figure 4:**
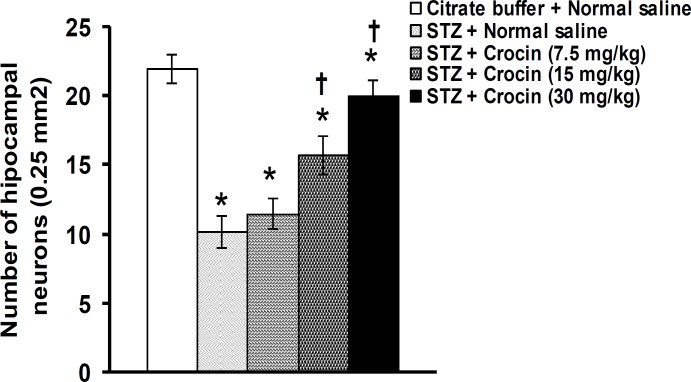
Effects of crocin on the number of hippocampal neurons in STZ-induced diabetic rats. Data are presented as mean±SEM (n= 6). *P<0.05 denotes significant difference *vs* citrate buffer + normal saline treated group. ^†^*P*<0.05 denotes significant difference *vs* STZ + normal saline treated group

**Figure 5:**
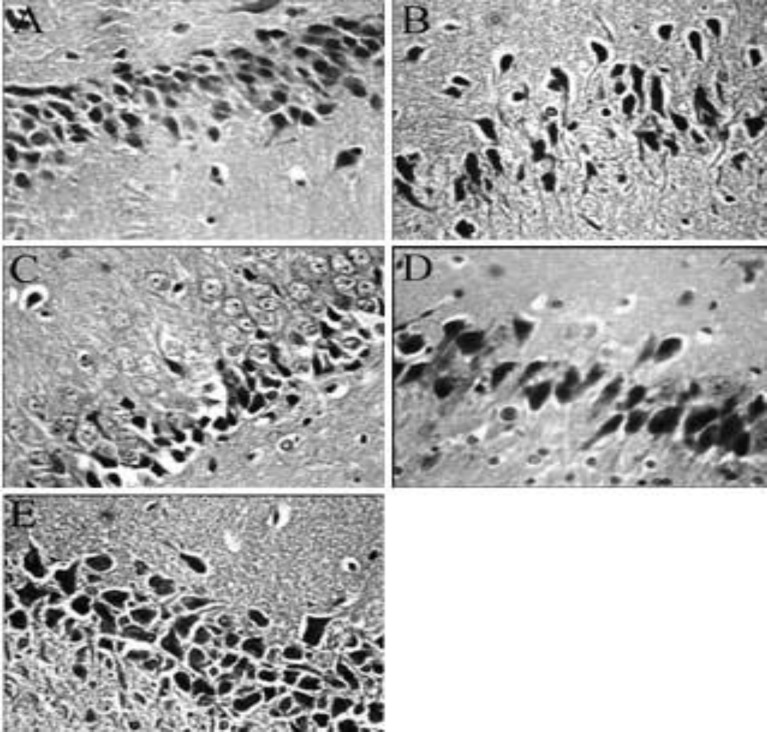
Histological analysis of CA1 region neurons of the hippocampus in rats belonging to different experimental groups. A: Citrate buffer + normal saline group, normal density of neurons in the hippocampus. B: STZ-induced diabetes, decreased number of neurons. C: STZ-induced diabetic rats treated with 7.5 mg/kg of crocin, shows no significant change compared to group B. D and E: STZ-induced diabetic rats treated with 15 and 30 mg/kg of crocin, respectively, the number and density of neurons increased (H&E ×400)

Figure 2 shows the effect of crocin on blood glucose and serum insulin changes induced by STZ in rats. Blood glucose significantly increased and serum insulin significantly decreased following IP injection of STZ. Crocin at a dose of 7.5 mg/kg had no significant effects, whereas 15 and 30 mg/kg of crocin significantly reversed the levels of blood glucose (F(4,25)= 23.477, *P*<0.05, Figure 3A) and serum insulin (F(4,25)= 32.813, *P*<0.05, Figure 3B) in diabetic rats. 

Figure 3 shows the effect of crocin on plasma TAC and MDA changes in STZ-induced diabetic rats. STZ decreased and increased the levels of plasma TAC and MDA, respectively. Crocin at a dose of 7.5 mg/kg produced no significant effects, whereas at doses of 15 and 30 mg/kg significantly recovered TAC (F(4,25)= 14.583, *P*<0.05, Figure 2A) and MDA (F(4,25)= 18.223, *P*<0.05, Figure 2B) levels in diabetic rats. 

Figures 4 and 5 show the effect of crocin on hippocampal neurons number in STZ-induced diabetic rats. As shown in Figures 3 and 4A, the number of neurons in the hippocampus was 22.5±1.3 in citrate buffer + normal saline treated group. STZ reduced the number of hippocampal neurons (Figures 3 and 4B). Crocin at a dose of 7.5 mg/kg did not prevent hippocampal neuron loss (Figures 3 and 4C). Crocin at doses of 15 and 30 mg/kg significantly prevented the decrease of neurons in the hippocampus (F(4,25)= 17.584, *P*<0.05, Figures 3, 4D, and 4E) of diabetic rats.

## Discussion

The present results showed that STZ impaired learning and memory by increasing TL_1_ and TL_2_ time durations and decreasing TLs percentage. Crocin improved STZ-induced learning and memory impairments by reversing TL_1_ and TL_2_ time durations and TLs percentage. It has been reported that TL measured on plus maze on the first day (TL_1_) serves as an index of learning and acquisition, whereas TL on the second day (TL_2_) serves as an index of retrieval and memory (38). A shortened TL, or not, during a maze re-exposure (LT_2_), relative to the first exposure (LT_1_) is indicative of good or impaired learning, respectively (39). STZ-induced diabetic rats showed higher LT_2_ than LT_1_ when examined 10 and 20 days after induction of diabetes (40). Da Cunha *et al* (30) showed that scopolamine and L-NAME produced amnesia by decreasing TLs percentage in EPM. In our study, the TL_2_ was not higher than TL_1_ in diabetic rats, but TLs percentage significantly decreased when compared with normal saline-treated group. Only in one study, STZ-induced diabetic rats showed cognitive deficits assessed using EPM test after eight weeks of induction of diabetes (41). Taken together, STZ produced memory and learning impairments using other learning and memory behavioral tasks in rats (5, 14, 42). There is no report describing the effects of crocin on learning and memory impairments in diabetic rats using EPM. Crocin prevented the inhibitory effect of ethanol on long-term potentiation (learning and memory physiologic phenomena) in the dentate gyrus and in the hippocampus of rats (43). In addition, memory and learning deficits induced by chronic stress and chronic cerebral hypoperfusion improved with long-term application of saffron and its active constituent, crocin in rats (10, 26). 

The results of the present study showed that STZ produced hyperglycemia and hypoinsulinemia. Crocin reversed STZ-induced changes in glucose and insulin levels. STZ is synthesized by Streptomycetes achromogenes and is used to induce diabetes in laboratory animals such as rats and mice (33). STZ enters B cells *via *a glucose transporter (GLUT2) using a variety of intracellular toxic mechanisms such as production of oxygen free radicals that causes degeneration of pancreatic B cells leading to hypoinsulinemia and subsequent hyperglycemia (33). The presence of insulin and insulin receptors in the hippocampus and cerebral cortex suggest a functional involvement in brain cognition phenomena such as learning and memory (11). Insulin deficiency is the initiating event that induces decreases in insulin signaling cascade in the brain and contributes to diabetes-induced cognitive deficits (44). Systemic treatment with insulin improved memory impairment in diabetic rats induced by streptozotocin (44, 45). Hyperglycemia plays a major role in diabetes-induced neuronal degeneration through increasing reactive oxygen species production and oxidative stress (3). Constant hyperglycemia (eight weeks) impaired learning and memory functions assessed by EPM in STZ-induced diabetic rats (41). The present study is the first report describing the protective effect of crocin on pancreas and inhibitory effect on subsequent events (hyperinsulinemia and hyperglycemia) in STZ-induced diabetic rats. Saffron, crocin, and safranal showed antihyperglycemic and antihypoinsulinemic effects in alloxan-induced diabetic rats (46). In addition, long-term IP injection of hydromethanolic extract of saffron produced hypoglycemia and hyperinsulinemia in healthy male rats (47). Saffron and crocin decreased neurotoxicity induced by high glucose in PC12 cells using as a suitable model of diabetic neuropathy (48).

The results of the present study showed that TAC decreased, but MDA increased in diabetic rats. Crocin reversed TCA and MDA changes induced by STZ. Oxidative stress, an imbalance between the generation of reactive oxygen species and antioxidant defense capacity of the body, is closely associated with diabetes and diabetic complications such as learning and memory deficits (49). Plasma TAC represents a suitable biochemical parameter for evaluating the overall antioxidant status resulting from antioxidant intake or production and their consumption by the increasing level of oxidative stress (50). Plasma MDA is a reliable and commonly used biomarker for assessing lipid peroxidation. Lipid peroxidation is a well-established mechanism of cellular injury and is frequently used as an indicator for oxidative stress in cells and tissues (51). It is well known that in STZ-induced diabetic rats, TCA, MDA, and antioxidative enzymes such as catalase were changed not only in plasma but also in other organs including kidney, liver, heart, and brain (49). In the STZ-induced diabetic rats, lipid peroxidation increased in the cerebral cortex, cerebellum, braistem, and learning and memory impaired in EPM (41). In the present study, plasma TAC and MDA instead of the hippocampus were measured, because other investigators have been measured oxidant/antioxidant activities in the brain areas including hippocampus in diabetic rats (49, 52). Crocin has a potent antioxidant property (53). The oxidative stress produced by chronic stress and chronic cerebral hypoperfusion in the hippocampus was reversed with IP injection of saffron and crocin (10, 26). Safranal, a constituent of saffron, attenuated cerebral ischemia-induced oxidative damage in rat hippocampus (54). 

In this study, STZ reduced the hippocampal neurons and crocin prevented STZ-induced neuron loss. The hippocampus is sensitive to diabetes and shows neuronal death (3). Zhang *et al* (11) reported neuronal proliferation and survival reduction in the hippocampus of streptozotocin-induced diabetic rats. STZ-induced diabetes produced a dramatic decrease in cell proliferation in the rat dentate gyrus as compared with controls (15). Insulin deficiency occurred in diabetes may have an important role in hippocampal neuron loss (55). Insulinomimetic C-peptide prevented hippocampal neuron loss in STZ-induced diabetic rats (56). Hyperglycemia plays a major role in diabetes-induced neuronal degeneration through increase in reactive oxygen species production and oxidative stress (3). STZ-induced diabetic hyperglycemia produced neuron death in various brain structures including cingulate cortex, thalamus nuclei, and hippocampus (56). Saffron and its constituent crocin have potent neuroprotective effects. Crocin and crocetin blocked the effect of lipopolysaccaride (LPS) on hippocampal cell death by reducing the production of intracellular reactive oxygen species (57). In addition, crocin enhanced nerve regeneration in sciatic nerve crush injury by its antioxidant activity (23).

## Conclusions

The results of the present study showed that streptozotocin impaired learning and memory functions in EPM through production of hypoinsulinemia, hyperglycemia, oxidative stress, and reduction of hippocampal neurons. Crocin improved STZ-induced learning and memory impairments by antihypoinsulinemic, antihyperglycemic, antioxidant, and neuroprotective effects. 
